# Clinical Efficacy of Fuzheng Guben Anticancer Decoction Combined with Taxol in Treating Ovarian Carcinoma and Its Effect on Complication Incidence

**DOI:** 10.1155/2021/2782875

**Published:** 2021-12-17

**Authors:** Pinger Li, Yinmei Lou

**Affiliations:** Integrated Traditional Chinese and Western Medicine, The First People's Hospital, Fuyang, Hangzhou 311400, Zhejiang, China

## Abstract

**Objective:**

To investigate the clinical value of Fuzheng Guben anticancer decoction combined with taxol in treating ovarian carcinoma (OC).

**Methods:**

The medical records of 80 OC patients treated in the First People's Hospital of Fuyang Hangzhou (January 2018–January 2021) were retrospectively analyzed, and the patients were split into the control group and the experimental group according to the treatment regimen, with 40 cases each. Those in the control group accepted the taxol chemotherapy, and on this basis, those in the experimental group took the Fuzheng Guben anticancer decoction, so as to compare its clinical efficacy and complication incidence.

**Results:**

No statistical between-group differences in patients' general information were observed (*P* > 0.05); compared with the control group, the disease objective remission rate of the experimental group was greatly higher (*P* < 0.05); before and after treatment, the changes in CD8^+^ were not significant, indicating no statistically significant between-group differences (*P* > 0.05), and after treatment, CD3^+^, CD4^+^, and CD4^+^/CD8^+^ were obviously higher than before and were obviously higher in the experimental group than in the control group (*P* < 0.05); after treatment, the CA125, CA199, and CEA levels were obviously lower than before and were significantly lower in the experimental group than in the control group (*P* < 0.05); the mean survival of the experimental group was significantly higher than that of the control group (19.80 ± 5.84 vs. 14.075 ± 5.12 months, *P* < 0.05); and between the two groups, the incidence rate of adverse reactions of the experimental group was remarkably lower (*P* < 0.05).

**Conclusion:**

On the basis of taxol chemotherapy, jointly applying Fuzheng Guben anticancer decoction can significantly improve the clinical efficacy of OC, help to improve patients' immune function, lower the complication incidence rate, and prolong the mean survival.

## 1. Introduction

Ovarian carcinoma (OC) is a malignant neoplastic disease that occurs in the ovaries of women, with an incidence second only to cervical cancer and endometrial carcinoma, which seriously threatens the life, health, and safety of women [1–4]. Clinically, the methods of treating OC mainly include surgical resection and chemoradiotherapy, and with the gradual and deep research of traditional Chinese medicine (TCM) in tumor treatment at the current stage, TCM is also applied for treating OC patients. Taxol can effectively inhibit tumor growth and control the condition, but chemotherapy will reduce the immunity, which, combined with the untoward drug effects, has a large effect on patients [5–8]. With the continuous development of TCM technology, Chinese herbs have been gradually used in treating OC as an adjuvant therapy to regulate body immunity and relieve the adverse reactions from taxol chemotherapy, thus having an important role in improving the clinical efficacy of OC [9–12]. Fuzheng Guben anticancer decoction has the efficacy of regulating qi flowing to invigorate blood, resolving hard mass for detumescence, invigorating qi for strengthening vital qi, and nourishing blood for consolidating body resistance, which is a good choice for the treatment of OC. At present, there are few studies on combining Fuzheng Guben anticancer decoction with taxol in treating OC patients. Based on this, the clinical efficacy of Fuzheng Guben anticancer decoction combined with taxol in treating OC and its effect on complication incidence were deeply analyzed in this study.

## 2. Study Methods

### 2.1. Patient Grouping

The medical records of 80 OC patients treated in the First People's Hospital of Fuyang Hangzhou from January 2018 to January 2021 were retrospectively analyzed, and the patients were divided into the control group and the experimental group according to whether they took the Fuzheng Guben anticancer decoction, with 40 cases each. Those only treated with taxol chemotherapy were included in the control group, and those who received the combined treatment of taxol chemotherapy and Fuzheng Guben anticancer decoction were included in the experimental group. The study was approved by the ethics committee of the First People's Hospital of Fuyang Hangzhou.

### 2.2. Inclusion Criteria

The inclusion criteria were as follows: ① the patients were diagnosed with OC after laboratory index inspection, imaging data, and pathological examination; ② the patients had complete data and high treatment compliance; ③ the patients met the indications of single taxol chemotherapy; and ④ the patients and their family members signed informed consent of the study.

### 2.3. Exclusion Criteria

The exclusion criteria were as follows: ① the patients had severe organ failure; ② the patients had cognition disorders; ③ the patients were allergic to the drugs applied herein; ④ the estimated survival of the patients was less than 6 months; and ⑤ the patients lost to follow-up during the study.

### 2.4. Methods

Single taxol chemotherapy was adopted to patients in the control group as follows. Before chemotherapy, the pretreatment with dexamethasone, diphenhydramine, and H2 receptor antagonist was performed; in the first 3 days of chemotherapy, 120–150 mg/mz of taxol (specification: 60 mg; manufacturing: Beijing Union Pharmaceutical Factory Co., Ltd.; NMPA approval no. H20083786) was administered via an intravenous drip, and at the 4^th^ day, 90 mg/mz of taxol was administered via abdominal cavity perfusion for 2-3 times daily. Four weeks were regarded as one course, and the patients were treated for 3 consecutive courses totally [13]. On this basis, the patients in the experimental group took the Fuzheng Guben anticancer decoction. The formula was 20 g of Chinese angelica, 20 g of debark peony root, 30 g of American ginseng, 30 g of Mongolian milk vetch root, 15 g of dried tangerine peel, 15 g of *Pinellia* tuber, 15 g of *Fritillaria*, 6 g of liquorice root, and 6 g of Chinese date. The herbs were decocted according to the general decocting method, and the patients daily took one dose in two split times (in the morning and the evening) when it was still warm. The follow-up time of all patients was not less than 12 months.

### 2.5. Observation Indicators

The patients' general information including their age, BMI, course of the disease, TNM stage, and pathological type was counted. The clinical treatment effect of patients in both groups was evaluated referring to the Response Evaluation Criteria in Solid Tumors (RECICT) [14] as follows. It was considered as complete response (CR) if the patients' lesion disappeared completely or for 4 weeks; partial response (PR) if the lesion was shrunk to over 30% for over 4 weeks; stable disease (SD) if the lesion was shrunk to less than 30% or increased by less than 20%; and progressive disease (PD) if the lesion was increased by more than 20% or there were new lesions. The objective remission rate (ORR) = (CR + PR)/total number × 100%.

The immune function indicators (CD3^+^, CD4^+^, CD8^+^, and CD4^+^/CD8^+^) were measured by the flow cytometry assay; and by drawing patients' upper limb venous blood, their levels of tumor markers including CA125, CA199, and CEA were measured by the enzyme-linked immunosorbent assay (ELISA), with the kits purchased from Shanghai Enzyme-linked Biotechnology Co., Ltd. Through follow-up visits, the patients' mean survival was recorded, and their adverse reactions were analyzed.

### 2.6. Statistical Processing

The data differences were calculated with SPSS 22.0, picture drawing software was GraphPad Prism 7 (GraphPad Software, San Diego, USA), the enumeration data and measurement data were expressed by (*n* (%)) and ($\bar{x}$ ± *s*) and examined by *X*^2^ test and *t*-test, respectively, and *P* < 0.05 indicated a statistical difference.

## 3. Results

### 3.1. General Information

After comparing the patients' general information including age, BMI, course of the disease, TNM stage, and pathological type, the result was *P* > 0.05, which indicated no great between-group difference and met the study criteria of the controlled experiment. See Table 1.

### 3.2. Clinical Efficacy

**Figure 1:**
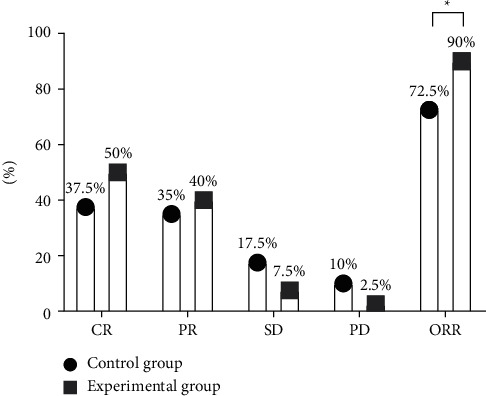
Between-group comparison of clinical efficacy. Note: the horizontal axis showed the evaluation dimensions, and the vertical axis showed the percentage. In the control group, there were 15 CR cases, 14 PR cases, 7 SD cases, and 4 PD cases, so the number of ORR cases was 29. In the experimental group, there were 20 CR cases, 16 PR cases, 3 SD cases, and 1 PD case, so the number of ORR cases was 36; ^*∗*^ indicated a significant between-group difference in ORR (*X*^2^ = 4.021, *P*=0.045).

Between the two groups, the ORR of the experimental group was greatly higher (*P* < 0.05). See Figure 1.

### 3.3. Immune Function

Before and after treatment, the variation in CD8^+^ of all patients was not significant, indicating no statistical meaning in the between-group difference (*P* > 0.05). After treatment, the patients' other immune function indicators were obviously higher than before (*P* < 0.05) and were significantly higher in the experimental group than in the control group (*P* < 0.05). See Table 2.

### 3.4. Levels of Tumor Markers

After treatment, the CA125, CA199, and CEA levels were obviously lower than before (*P* < 0.05) and were significantly lower in the experimental group than in the control group (*P* < 0.05). See Table 3.

### 3.5. Long-Term Efficacy

**Figure 2:**
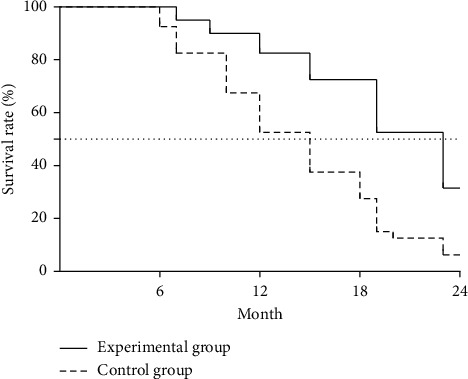
Survival curves.

The mean survival of the experimental group was significantly higher than that of the control group (19.80 ± 5.84 vs. 14.075 ± 5.12 months, *t* = 4.544, *P* < 0.001). See Figure 2 for the survival curves.

### 3.6. Adverse Reactions

Between the two groups, the incidence rate of adverse reactions in patients was significantly lower in the experimental group (*P* < 0.05). See Table 4.

## 4. Discussion

With the development of China's society and economy, people's standard of living has been increasing and so has the life, work, and other stresses in women, and combined with objective factors such as environmental pollution, the number of new OC cases is rising year by year, and more and more young women also suffer from the disease, which has a fatality rate ranking the first among malignant tumors of the female reproductive system [15–18]. Currently, chemotherapy is the main means for the treatment of cancer, mainly improving patient symptoms and prolonging the survival through Western medicine [19–22]. Taxol is a natural anticancer drug extracted from Chinese yew. It exerts antitumor effects by interfering with the microtubule network essential for cell function in mitosis and interphase and can inhibit cell-cycle microvascular protein depolymerization, cell division, and consequently cell metabolism; in addition, it is metabolized through the liver, has little renal toxicity, and is significantly less toxic than platinum-based chemotherapy. In the study conducted by Guoqiang et al. [23] and Jiwon et al. [24], taxol has better antitumor effect in diseases such as breast cancer, lung cancer, and ovarian cancer. From the perspective of TCM, OC belongs to the category of “abdominal mass,” which is mostly caused by weakness of the body, insufficient essence and blood of the liver and kidney, internal binding of phlegm turbidity and stasis toxin, etc., and chemotherapy can cause loss of yin, yang, blood, and qi, blood flow blockage, and weak organs in patients and even symptoms such as incoordination between the spleen and stomach and qi-blood loss in some cases. TCM believes that treating OC shall comply with the principle of strengthening body resistance and restoring vital energy, which shall focus on both warming the kidney and promoting blood circulation. In this study, the Fuzheng Guben anticancer decoction contained Chinese angelica (nourishing yin for regulating menstruation and promoting blood circulation for relieving pain), debark peony root (tonifying blood for consolidating body resistance), American ginseng and Mongolian milk vetch root (nourishing the spleen for invigorating qi and warming the kidney for reinforcing yang), dried tangerine peel (regulating qi flowing for invigorating the spleen and regulating the spleen-stomach for dispelling dampness), *Pinellia* tuber (relieving oppression for resolving hard mass), *Fritillaria* (resolving masses for detumescence), liquorice root (clearing heat and removing toxicity, invigorating the spleen-stomach, and replenishing qi), and Chinese date (regulating the spleen-stomach and nourishing qi, tonifying blood, and invigorating the spleen). Combining all the herbs could exert the efficacy of replenishing qi, tonifying blood, invigorating the spleen and stomach, and warming the kidney for reinforcing yang. This formula is quite valued in the field of TCM against cancer, and a large number of animal experimental studies have been done on its anticancer effects in the international medical community. It was reported that, in a set of induced bladder cancer experiments, rats fed with Fuzheng Guben anticancer decoction were in a better state than the others, and this formula also showed a marked effect on liver cancer metastasis in experimental mice, presenting less side effects, the function of enhancing the immune function of patients, and an anticancer role.

In this study, the ORR was greatly higher in the experimental group than in the control group (*P* < 0.05), which was consistent with the study report of Yiming and Huijun [25], implying that applying Fuzheng Guben anticancer decoction on the basis of taxol chemotherapy could effectively improve the efficacy of OC and was good for controlling the condition. Before and after treatment, the variation in CD8^+^ of all patients was not significant, with no statistically meaningful between-group difference (*P* > 0.05); after treatment, the patients' other immune function indicators were obviously higher than before and were significantly higher in the experimental group than in the control group (*P* < 0.05). OC patients are often accompanied by immune dysfunction, which in turn is one of the important factors leading to disease progression, and among the immune function indicators, CD4^+^ in T lymphocytes can assist other immune cells to exert antitumor effects. This result suggested that the combined regimen could greatly strengthen the immunity and promote the recovery of OC patients, and the reasons were as follows: Fuzheng Guben anticancer decoction could alleviate the damage of taxol chemotherapy on patients' immunity, reduce the body damage, and protect patients' immune function; modern pharmacology indicated that astragalin and saponin from Mongolian milk vetch root could promote the body to synthesize proteins, improve immunity, and enhance cell antitumor activity; in addition, American ginseng was rich in polysaccharide, which could induce tumor cell apoptosis. After treatment, the patients' CA125, CA199, and CEA levels were obviously lower than before and were significantly lower in the experimental group than in the control group (*P* < 0.05), demonstrating that Fuzheng Guben anticancer decoction could effectively reduce the high expression of tumor markers in pathological tissue and serum of OC patients. According to the analysis on long-term efficacy, compared with the control group, the experimental group achieved significantly higher mean survival (19.80 ± 5.84 vs. 14.075 ± 5.12 months, *P* < 0.05) and lower incidence rate of adverse reactions (*P* < 0.05), implying that Fuzheng Guben anticancer decoction could consolidate body resistance, cultivate the primordial spirit, and effectively reduce the adverse reactions caused by taxol.

In conclusion, jointly applying Fuzheng Guben anticancer decoction on the basis of taxol chemotherapy significantly improves the clinical efficacy, promotes immune function, reduces the incidence of complications, and prolongs the mean survival. However, because the sample size of this study was small, the conclusions obtained herein still need to be confirmed by a large-sample randomized double-blind trial; the study only intervened some patients who met the indication of single taxol chemotherapy, so it has certain limitations, and the effect of combining the TCM formula with other chemotherapeutic agents still needs to be explored.

## Figures and Tables

**Table 1:** General information (*n* = 40).

Observation indicator	Control	Experimental	*X* ^2^/*t*	*P*
Age (years)	51.17 ± 8.58	51.30 ± 8.79	0.067	0.947
BMI (kg/m^2^)	23.24 ± 2.11	23.15 ± 2.08	0.192	0.848
Course of the disease (years)	2.27 ± 0.30	2.31 ± 0.32	0.577	0.566
TNM stage				
IIIa	18 (45%)	21 (52.5%)	0.450	0.502
IIIb	14 (35%)	13 (32.5%)	0.056	0.813
IV	8 (20%)	6 (15%)	0.346	0.556
Pathological type				
Serous cystadenocarcinoma	12 (30%)	13 (32.5%)	0.058	0.809
Mucinous cystadenocarcinoma	10 (25%)	8 (20%)	0.287	0.592
Granular cell carcinoma	7 (17.5%)	8 (20%)	0.082	0.775
Embryonal carcinoma	7 (17.5%)	6 (15%)	0.092	0.762
Undifferentiated carcinoma	4 (10%)	5 (12.5%)	0.125	0.723

**Table 2:** Between-group comparison of T-cell subset levels ($\bar{x}$ ± *s*).

T-cell subset		Control	Experimental	*t*/*P*
CD3^+^ (%)	Before treatment	46.55 ± 5.54	46.76 ± 5.60	
	After treatment	52.03 ± 5.84^*∗*^	57.81 ± 6.02^*∗*^	4.359/<0.001

CD4^+^ (%)	Before treatment	32.74 ± 4.82	32.86 ± 4.85	
	After treatment	37.13 ± 4.30^*∗*^	41.60 ± 4.76^*∗*^	4.407/<0.001

CD8^+^ (%)	Before treatment	32.60 ± 3.29	32.44 ± 3.18	
	After treatment	33.82 ± 3.19	33.86 ± 3.21	0.056/0.956

CD4^+^/CD8^+^	Before treatment	1.03 ± 0.03	1.04 ± 0.04	
	After treatment	1.10 ± 0.05^*∗*^	1.25 ± 0.05^*∗*^	13.416/<0.001

^*∗*^indicated *P* < 0.05 in the comparison of patients in the same group before and after treatment.

**Table 3:** Between-group comparison of levels of tumor markers ($\bar{x}$ ± *s*).

Test indicator		Control	Experimental	*t*/*P*
CA125 (U/ml)	Before treatment	90.86 ± 14.55	90.57 ± 14.33	
	After treatment	55.83 ± 10.62^*∗*^	33.02 ± 10.13^*∗*^	9.829/<0.001

CA199 (IU/L)	Before treatment	50.86 ± 6.64	51.02 ± 6.71	
	After treatment	30.91 ± 4.17^*∗*^	21.75 ± 4.39^*∗*^	9.568/<0.001

CEA (ng/ml)	Before treatment	27.25 ± 4.88	27.17 ± 4.79	
	After treatment	20.35 ± 3.22^*∗*^	16.02 ± 3.07^*∗*^	6.155/<0.001

^*∗*^indicated *P* < 0.05 in the comparison of patients in the same group before and after treatment.

**Table 4:** Adverse reaction incidence (*n* (%)).

Adverse reaction	Control	Experimental	*X* ^2^	*P*
Neurotoxicity	2 (5)	0 (0)		
Reduction of the white blood cell count	3 (7.5)	1 (2.5)		
Nausea and vomiting	6 (15)	3 (7.5)		
Anemia	4 (10)	0 (0)		
Joint and muscle pain	5 (12.5)	1 (2.5)		
Thrombocytopenia	4 (10)	2 (5)		
Total incidence rate	24 (60)	7 (17.5)	15.221	<0.001

## Data Availability

The data used to support the findings of this study are available from the corresponding author upon reasonable request.
